# Supplementary material for: Two-dimensional topological superconductivity in Pb/Co/Si(111)
Source: Nat Commun. 2017 Dec 11;8:2040. doi: 10.1038/s41467-017-02192-x (PMC5725560; doi:10.1038/s41467-017-02192-x)
Supplement: Supplementary file 1 — Supplementary Information [file 41467_2017_2192_MOESM1_ESM.pdf]

SUPPLEMENTARY NOTE 1: DECONVOLUTION OF THE SUPERCONDUCTING GAP OF THE TIP

The experimental set of data presented here was obtained by using a superconducting tip in order to increase the energy resolution. This tip was prepared from a Pt-Ir wire that was covered with Pb by crashing the tip into 3D islands of Pb grown over SiC clusters. The gap of the tip was measured at the end of each conductance map at the temperature $T = 2.05$ K which is above the superconducting critical temperature $T_c = 1.8$ K of the SIC Pb/Si(111) monolayer. The conductance spectra were fitted assuming a BCS gap with a finite pair breaking. More precisely we used the Usadel equations for a diffusive superconductor with a gap $\Delta_{\mathrm{tip}} = 1.41$ meV and a pair-breaking parameter $\Gamma_{\mathrm{Usadel}} = 0.001$ meV, see supplementary figure 1. We use the complex $\theta(E)$ representation of Usadel equation :$iE\sin(\theta) + \Delta\cos(\theta) - \Gamma = 0$ in which the DOS is given by $N(E) = N_0\Re(\cos(\theta))$. The pair breaking is almost negligible, hence the tip DOS is almost perfectly BCS like. The pair breaking parameter thus mainly served to avoid the BCS singularity in the numerical calculations. We found that the tip superconducting gap stayed constant at 1.41 ± 0.02 meV during our whole measurement run. This gap value is similar to the one of bulk Pb (Pb being a multigap superconductor [4] the tunneling gap varies from 1.35 to 1.5 meV depending on the band that contributes to the tunneling). As shown below, an independent crosscheck was done by measuring the conductance as a function of temperature with both the sample and the tip in the superconducting state. The temperature dependence allows us to precisely measure the tip and sample gap by fitting the thermal peak.

The conductance maps shown in figures 1 and 2 of the main text were measured at $T = 300$ mK. At such temperature both the tip and the sample are superconducting, and we are in the so-called S-I-S configuration. In this case there is no direct relation between the $\mathrm{d}I/\mathrm{d}V$ conductance spectra and the local density of states (LDOS) of the sample. The conductance is a convolution of the tip and sample density of states. Due to the gap of the tip, a feature that appears at the energy E in the sample LDOS will appear shifted at the voltage $eV \approx E + \Delta_{\mathrm{tip}}$ in the S-I-S conductance spectra. Thus, in order to recover the density of states of the sample, one has to deconvolute the conductance from the tip DOS. The simplest way to do that is to suppress a window of size $2\Delta_{\mathrm{tip}}$ from the conductance curve. This basic method is illustrated on supplementary figure 6. The suppressed part is shown

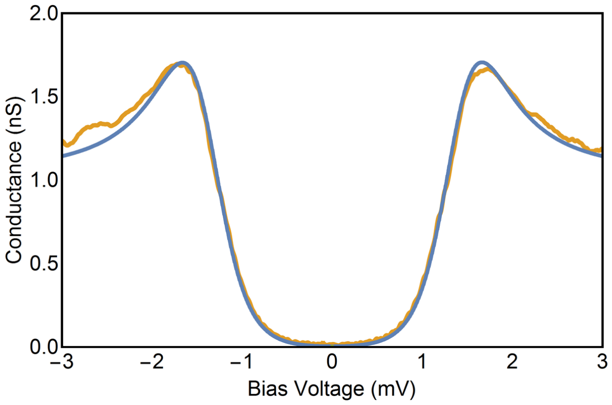

Supplementary Figure 1: **Tunneling gap of the Pb tip** - The yellow curve is an experimental tunneling spectrum measured at 2.05 K, i.e. above the critical temperature of the Pb/Si(111) monolayer. In blue, fit with Usadel theory with a gap $\Delta_{\text{tip}} = 1.41$ meV and a pair breaking parameter $\Gamma_{\text{Usadel}} = 0.001$ meV.

in between the dotted lines. This crude method gives a good qualitative picture of the edge states but it cannot give the correct LDOS of the sample. However, a precise determination of the sample LDOS can be obtained by using an inversion method to deconvolute the sample and tip DOS as we describe below.

In SIS spectroscopy the tunneling current is given by:

$$I(V) \propto \int_{-\infty}^{\infty} \mathrm{d}E N_{\text{tip}}(E) N_{\text{sample}}(E + eV) \left(f_D(E) - f_D(E + eV)\right) \tag{1}$$

Where N_{tip} and N_{sample} are respectively the LDOS of the tip and of the sample and $f_{\text{D}}(E)$ is the Fermi-Dirac distribution. In practice we find that we need to take into account and additional Lorentzian broadening of half-width-at-half-maximum $L = 28$ μeV that can originate from several sources: external RF noise (jitter) or dynamical Coulomb blockade (DCB). If the charging energy of the tunneling electron exceeds the thermal energy, one cannot neglect the DCB which is usually described by the $P(E)$ theory. The capacitance of the tunnel junction mediates an interaction of the tunneling electrons with the surrounding electromagnetic environment that leads to a broadening of the tunneling spectra [1, 2]. Due to the Lorentzian broadening of the tunneling spectra the energy resolution of our experiments is limited to ≈ 30 μeV.

The deconvolution procedure assumes that the DOS of the tip is given by a BCS-Usadel shape with $\Delta_{\text{tip}} = 1.41$ meV and a pair-breaking parameter $\Gamma_{\text{Usadel}} = 0.001$ meV as given

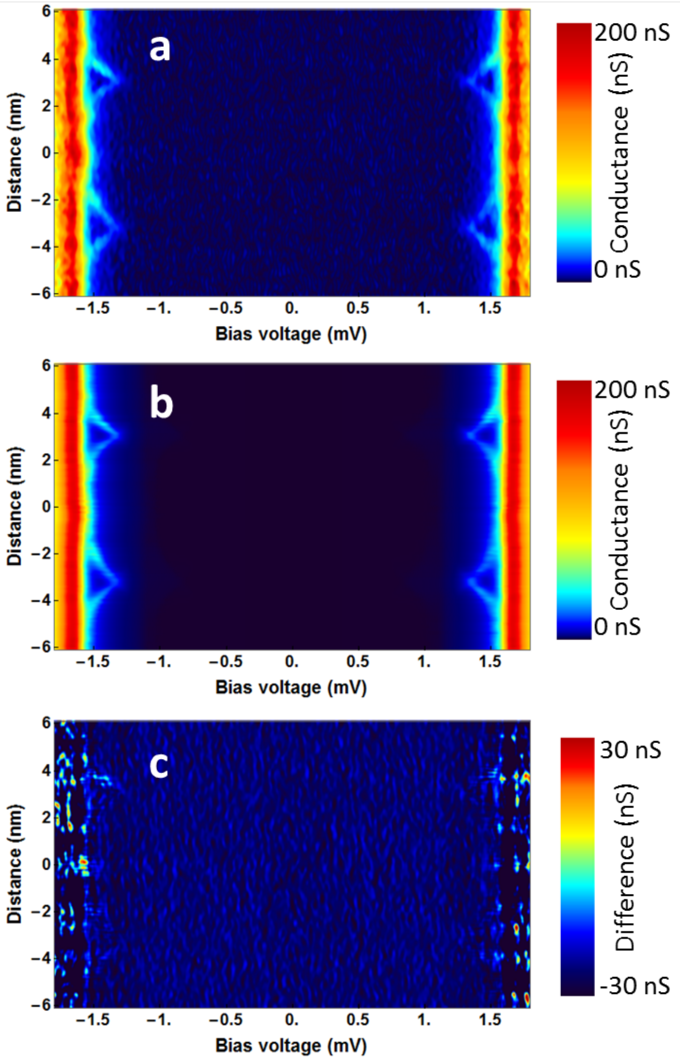

Supplementary Figure 2: **Deconvolution procedure of raw data - a.** Color coded representation of the S-I-S tunneling spectra taken along a line cut passing through the magnetic domain (see figure 2 of main text). **b.** Fit of the tunneling spectra along a linecut with the procedure described in the text (For the sample the LDOS is approximated by a BCS-Usadel gap and two gaussian in-gap peaks, the tip DOS is given by a BCS-Usadel gap). **c.** Difference between the raw data and the fit showing that the fitting procedure is very efficient to account for the in-gap states.

by the fit of the DOS at 2.05 K. Then we assume that the sample LDOS is the sum of a BCS-Usadel gap and two gaussian peaks at energy $\pm E_{\text{peak}}$ of amplitude A_+ and A_- and width σ_+ and σ_-. The fitting procedure showed that the BCS-Usadel gap was of constant amplitude $\Delta_{\text{sample}} = 0.293$ meV and we let Γ_{tip} as a free parameter. Some typical fits with

this procedure are shown in figure 2f and 2g of the main text. Figure 2e was obtained by deconvoluting 200 spectra taken along a linecut. The whole set of raw spectra used for supplementary figure 2e is shown on supplementary figure 2.a and the corresponding fits are shown on supplementary figure 2.b. The sample LDOS deduced from the fitting procedure is shown on figure 2e of main text. There is a good qualitative agreement between the fit and the data. A quantitative test can be done by subtracting the fitted spectra from the data as shown on supplementary figure 2c. As one can see, the in-gap states signature is almost suppressed in the difference image which means that they are perfectly fitted.

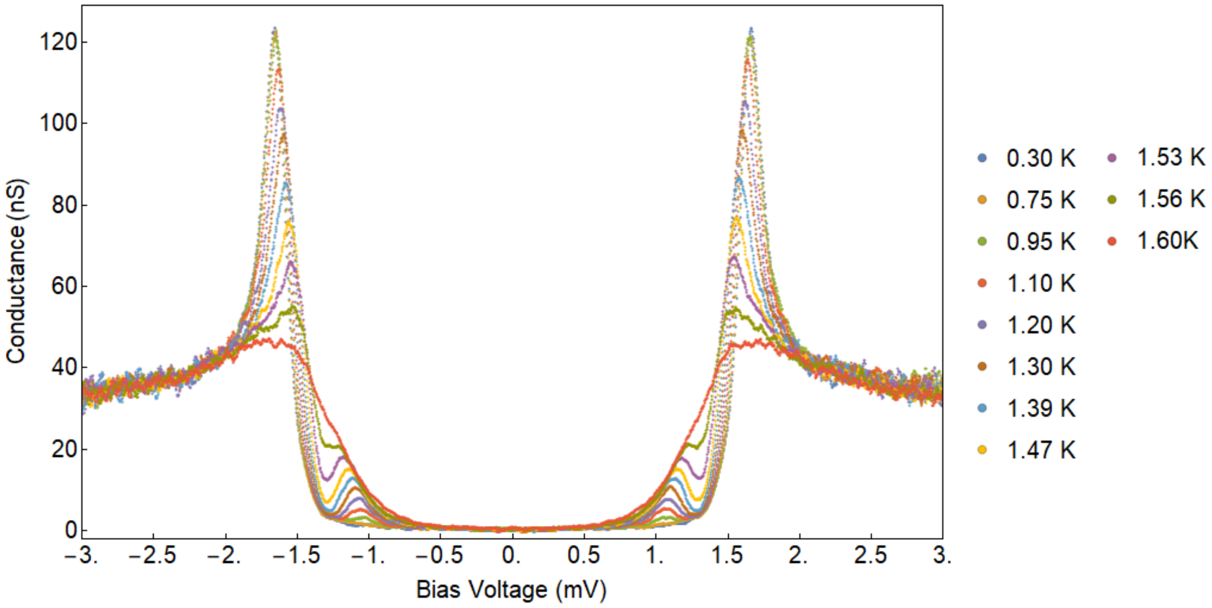

Supplementary Figure 3: **SIS conductance as function of temperature** - Conductance spectra obtained with a superconducting Pb tip and the Pb/Si(111) sample when the temperature is raised from 300 mK to 1.6 K. At 1.6 K the gap of the sample is closed and the conductance exhibits the BCS gap of the tip.

The deconvolution procedure is dependent on the precise energy that we extract for the superconducting tip. If the gap of the tip was taken much smaller than 1.41 meV, then the deconvoluted energy dispersion of the in-gap states could potentially show an anticrossing. Thus it is important to verify that the gap values used to fit the tip and sample DOS are correct to a precision of the order of 10 μeV. In order to do that, one can measure the S-I-S conductance as function of temperature far from any magnetic domain.

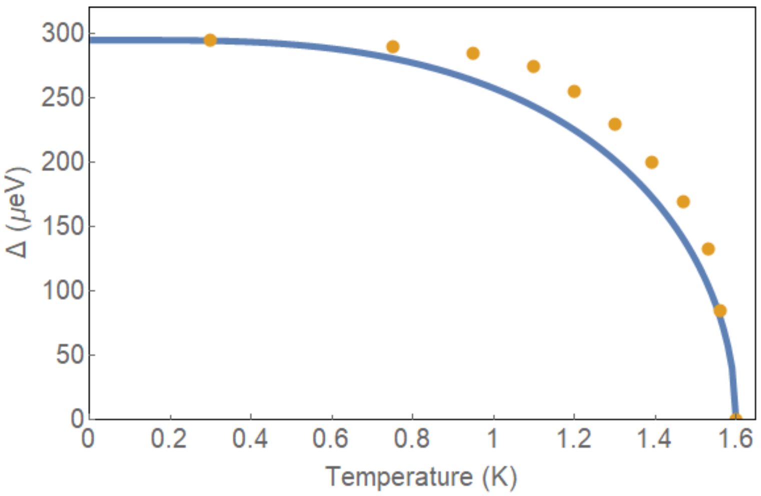

Supplementary Figure 4: **Deconvolution procedure of raw data** - Gap of the Pb/Si(111) monolayer as function of temperature (yellow dots) determined by fitting the conductance curves of supplementary figure 3. The blue line is the BCS gap dependence for a zero temperature gap of 0.295 meV and a critical temperature of 1.6 K.

Supplementary figure 3 shows an ensemble of S-I-S spectra between the Pb tip and the SIC-Pb/Si(111) sample from 300 mK to 1.6 K where the sample gap closes. When the temperature is rising a so-called thermal peak appears inside the gap at a characteristic energy $\Delta_{\text{tip}} - \Delta_{\text{sample}}$ as demonstrated by Ivar Giaver in 1960 [3]. At 300 mK one sees only a conductance peak at $\Delta_{\text{tip}} + \Delta_{\text{sample}}$ but above 700 mK a small peak at $\Delta_{\text{tip}} - \Delta_{\text{sample}}$ starts to appear. The amplitude of this peak increases exponentially with temperature up to the critical temperature of the Pb monolayer. Note that for a non-BCS gap, like the Usadel gap that we use, the positions of the peaks are slightly shifted by the pair breaking parameter Γ. In order to get precise gap values we fitted the S-I-S conductance with the model explained above and we obtained the temperature dependence of the sample as shown on supplementary figure 4. These measurements lead to $\Delta_{\text{tip}} = 1.41$ meV and $\Delta_{\text{sample}} = 0.295$ meV with the sample at 300 mK. The critical temperature of the lead monolayer is estimated to be 1.60 K.

SUPPLEMENTARY NOTE 2: ANOTHER EXAMPLE OF EDGE STATE

In supplementary figure 5 we present another example of edge states observed experimentally in a sample different from the one presented in the main text. In this configuration we recover the same basic characteristic states crossing the superconducting gap as shown on supplementary figure 6. In this case, the underlying Co cluster was elongated while the one from the main text was almost circular. As a result, the edge states we image are also elongated.

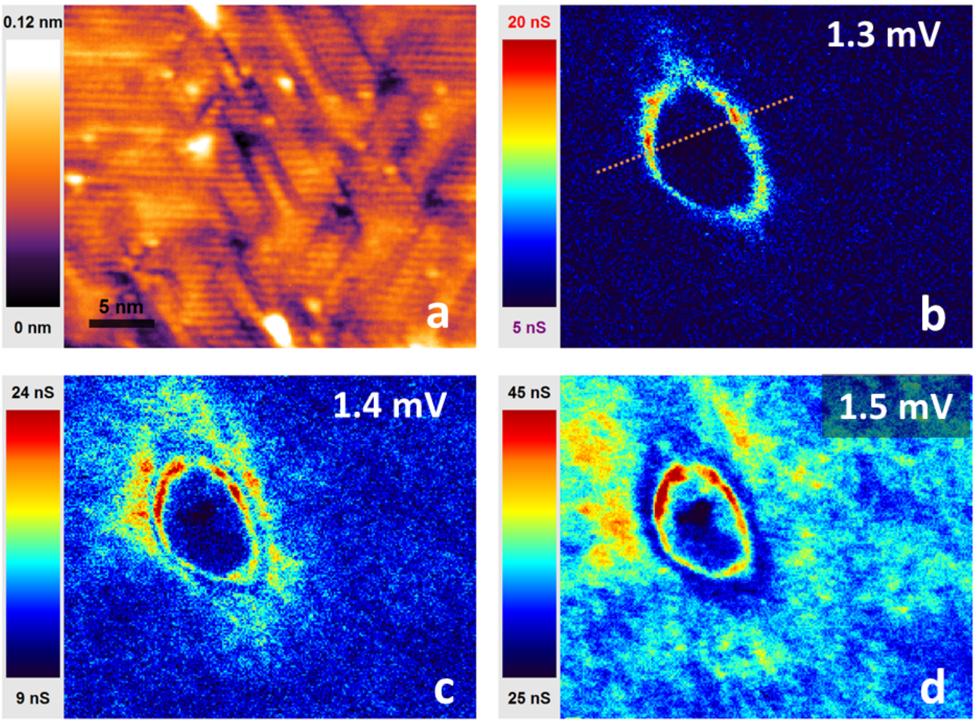

Supplementary Figure 5: **Measurement of other topological edge states - a**. Topography of an area of the SIC Pb/Si(111) monolayer including a topological domain. **b-d**. In-gap conductance maps of the same area showing the spatially dispersing topological edge states.

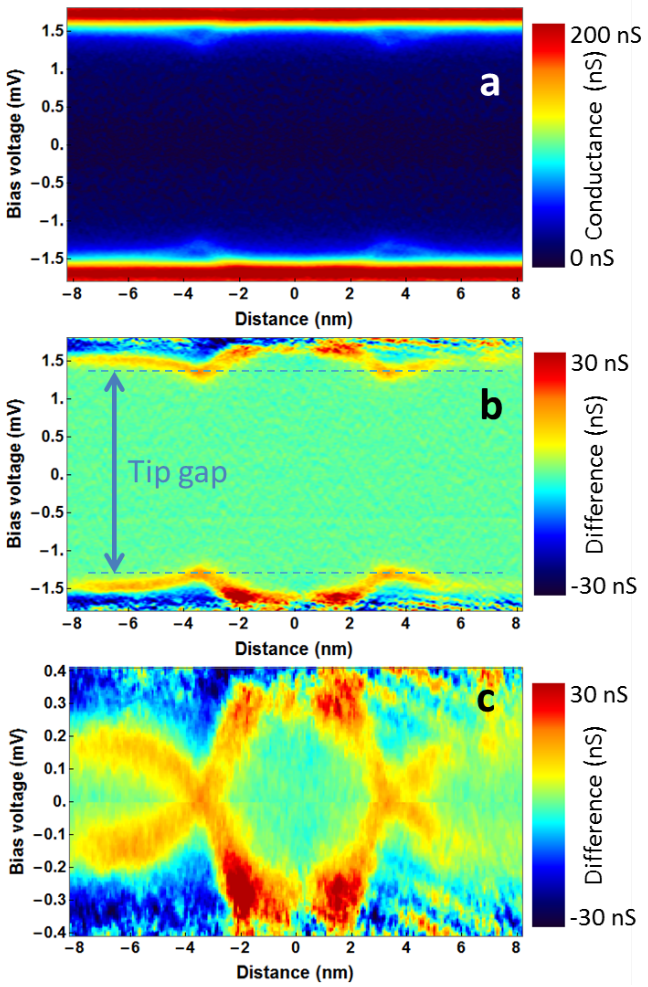

Supplementary Figure 6: **Spectra taken along a line passing through a topological domain - a**. Energy- and space-dependent line cut through the dashed orange line indicated on supplementary figure 5b showing in gap states. **b**. In order to improve the contrast, a reference spectrum, taken far from the magnetic domain, was subtracted to all the conductance spectra along the linecut. The in-gap states appear quite clearly in yellow. Due to the use of a Pb superconducting tip, all the states appear shifted by $\approx \Delta_{\mathrm{tip}}$. The blue dashed lines indicates the typical energy shift due to the Pb tip. **c**. In order to get a qualitative idea of the LDOS of the sample one can compensate for the energy shift induced by the Pb tip by suppressing the energy window in-between the two dashed lines shown in b. The obtained figure thus clearly shows the edge states having a X-shaped crossing. This is very similar to the one pictured on figure 2e of the main text where a more precise deconvolution procedure was used.

SUPPLEMENTARY NOTE 3: PHASE DIAGRAM OF TOPOLOGICAL SUPERCONDUCTIVITY

In order to draw the phase diagram of our system, we will discuss in more details the bulk Hamiltonian describing the topological superconductor. In the Main Text we depicted our minimal model accounting for the effects observed in the experiment, and here we cast this Hamiltonian in the following condensed form:

$$H_{\text{Top}} = \sum_k \Psi_k^\dagger H(k) \Psi_k \,, \tag{2}$$

where the Nambu basis spinor is given by $\Psi_k^\dagger = \left(\hat{c}_{k\uparrow}^\dagger,\ \hat{c}_{k\downarrow}^\dagger,\ \hat{c}_{-k\downarrow},\ -\hat{c}_{-k\uparrow}\right)$ and

$$H = \xi_k \tau_z + \Delta_S \tau_x + V_z \sigma_z + \left(\alpha \tau_z + \frac{\Delta_{\text{T}}}{k_{\text{F}}} \tau_x\right)(\sigma_x k_y - \sigma_y k_x)\,. \tag{3}$$

with $\xi_k = k^2/2m - \mu$. τ_j and σ_j (with $j = x, y, z$) are Pauli matrices acting in the particle-hole and the spin space respectively. V_z is the Zeeman splitting, and we use $k_{\text{F}} = \sqrt{2m\mu}$ in order to express Δ_{T} in the same energy units as Δ_{S}.

In a spatially invariant case (for $V_z(r) = V_z = \text{cst}$), this 4×4 matrix can easily be diagonalized in order to obtain the eigen-energies of the system. We find four solutions: two electron-like and two hole-like with symmetric energies with respect to the Fermi level. These are given by the following expression:

$$E_\pm^2(k) = V_z^2 + (\alpha k)^2 + \Delta_S^2 + \Delta_{\text{T}}^2 \frac{k^2}{k_{\text{F}}^2} + \xi_k^2 \pm 2\sqrt{V_z^2(\Delta_S^2 + \xi_k^2) + \frac{k^2}{k_{\text{F}}^2}\left(\Delta_{\text{S}}\Delta_{\text{T}} + \alpha k_{\text{F}} \xi_k\right)^2}\,. \tag{4}$$

From this dispersion relation it becomes possible to compute the points at which the gap will close (where the system actually undergoes a topological transition) before reopening as a function of the different parameters. It is worth mentioning that deviations of the magnetic field orientation from the z axis only results in replacing V_z^2 with $V_z^2 + V_\parallel^2$, with $V_\parallel$ being the in-plane component of the magnetic field. Thus, the resulting spectrum and the phase diagram are robust against changes in the magnetic field orientation, and only depend on its strength. Supplementary figure 7 presents two phases diagram calculated in the limits $\Delta_{\text{T}} = 0$ and $\alpha = 0$ respectively.

In the first case, namely for $\Delta_{\text{T}} = 0$, the dispersion is given by:

$$E_\pm^2(k) = V_z^2 + (\alpha k)^2 + \Delta_{\text{S}}^2 + \xi_k^2 \pm 2\sqrt{V_z^2(\Delta_{\text{S}}^2 + \xi_k^2) + (\alpha k \xi_k)^2}\,, \tag{5}$$

and the system undergoes a topological transition from a trivial superconductor to a chiral one at a critical field

$$V_{z,\mathrm{crit}} = \sqrt{\Delta_{\mathrm{S}}^2 + \mu^2} \tag{6}$$

where the gap closes at $k = 0$. It is worth noting here that even though the spin-orbit coupling does not explicitly intervene in the expression of the critical field, its presence is absolutely necessary to guarantee the reopening of the gap at larger fields. It is easily seen that for $\alpha = 0$, the gap does not reopen and thus prevents the appearance of a *gapped* topological superconducting phase. The corresponding figures are shown in supplementary figures 7a,c.

The second case discussed in supplementary figures 7.b,d corresponds to the case $\alpha = 0$ and $V_z = 0$ for which the superconductor is time-reversal invariant, and we expect a transition from a trivial to a helical state. In this case, the topological transition is controlled by the amplitude of the triplet term Δ_{T}. From supplementary equation 4 we can write in the helical case

$$E_{\pm}^2(k) = \left(\Delta_{\mathrm{S}} \pm \frac{|k|}{k_{\mathrm{F}}}\Delta_{\mathrm{T}}\right)^2 + \xi_k^2. \tag{7}$$

The topological transition is thus obtained for $\Delta_{\mathrm{S}} = \Delta_{\mathrm{T}}$ at $k = k_{\mathrm{F}}$ and is represented in supplementary figure 7.d.

By introducing both a triplet order parameter and a Zeeman field, one can tune the transition line between the trivial and the helical states. This transition point can be found from supplementary equation 4 as

$$\Delta_{\mathrm{T}} = \sqrt{\Delta_{\mathrm{S}}^2 - V_z^2}\,, \tag{8}$$

with the gap closing happening in this case at $k = k_{\mathrm{F}}$.

The trivial to topological transition is slightly modified when spin-orbit interaction is included. The transition point in the k space is slightly moved away from k_{F}, and a simple analytical expression for the transition line is not accessible anymore. Nevertheless, this curve can be calculated numerically and it is plotted in supplementary figure 8 and Fig. 3a of main text for a Rashba interaction $k_{\mathrm{F}}\alpha = 1.7$ and a singlet order parameter $\Delta_{\mathrm{S}} = 3$. The only effect spin-orbit has on the transition line is to distort the trivial to helical transition line at large V_z as well as to increase the value of Δ_{T} for which the zero field helical transition occurs.

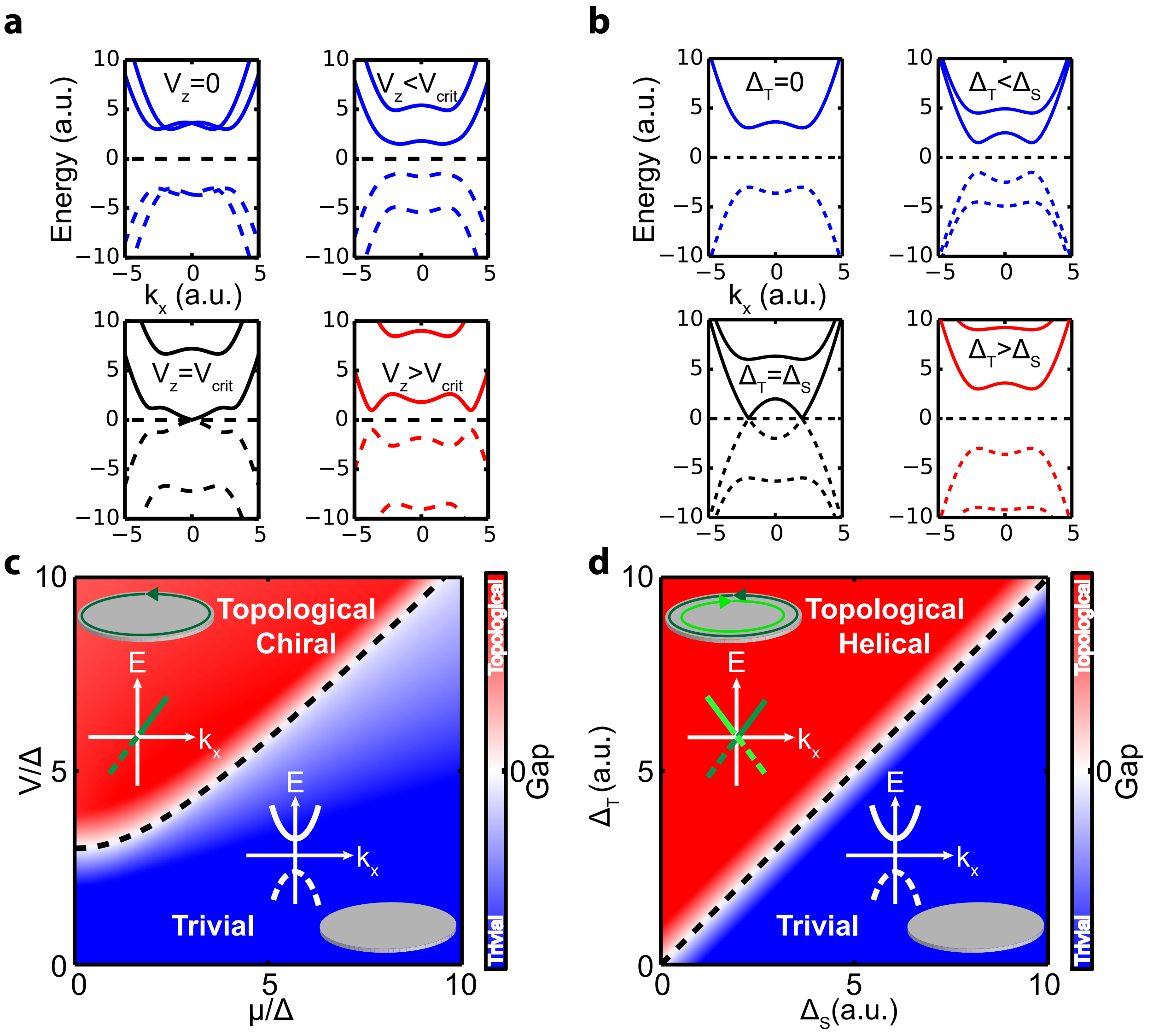

Supplementary Figure 7: **Phase diagrams for topological superconductivity - a.** Evolution of the band structure of an s-wave superconductor with Rashba spin-orbit coupling for different values of the Zeeman interaction. The black dashed line indicates the transition between the topological and trivial regimes. **b.** Evolution of the band structure of a superconductor for increasing values of the amplitude of the p-wave component. **c.** Phase diagram of the system parametrized by the chemical potential μ and the strength of the Zeeman coupling V.**d.** Phase diagram of the system parametrized by the amplitude of the singlet (Δ_S) and triplet (Δ_T) order parameters. The black dashed lines in c and d indicate the transitions between the topological and trivial regimes.

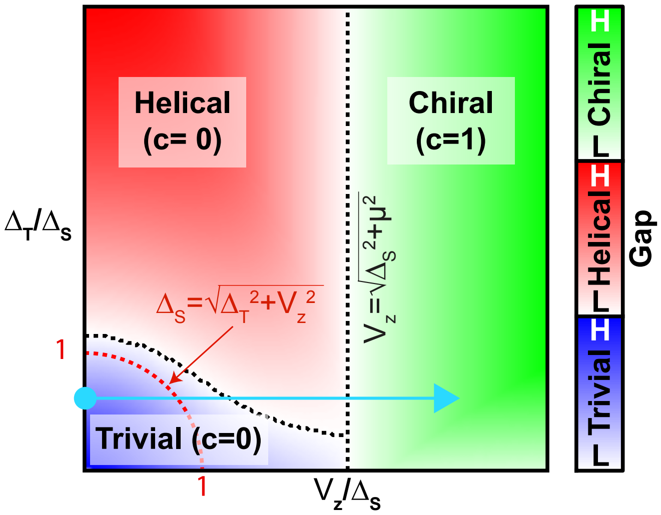

Supplementary Figure 8: **Phase diagram of topological superconductivity with the inclusion of spin-orbit interaction** - This diagram uses the Hamiltonian from which Fig 3.a. from the main text was obtained. Compared to the previous plots, we have added here the spin-orbit interaction that only modifies one of the transition line (drawn as a red dashed line) in order to make it merge with the chiral transition line. The parameters value used here are $\Delta_\mathrm{S} = 3.$ and $k_\mathrm{F}\alpha = 1.7$ with V_z and Δ_T ranging from 0 to $4\Delta_\mathrm{S}$. The red dashed line corresponds to the position of the topological transition in the case $\alpha = 0$.

SUPPLEMENTARY NOTE 4: CHERN NUMBER

The bulk spectrum along with the gap closing points (or lines) does not suffice to account for the number of edge states in a system with boundaries occurring in each phase. For that, we calculate the Chern number for each phase (the symmetry being chiral, a Chern number fully describes the number of edge states). The Chern number is defined as the integral curvature of the filled band $n(k)$, which reads:

$$\begin{aligned} C &= \sum_n \frac{1}{2\pi i} \int_{\mathrm{BZ}} \mathrm{d}^2 k F_n(k) \\ F_n(k) &= \frac{\partial A_{ny}(k)}{\partial x} - \frac{\partial A_{nx}(k)}{\partial y} \\ A_{n\mu}(k) &= \langle n(k) | \partial_\mu | n(k) \rangle \,, \end{aligned} \tag{9}$$

where $F_n(k)$ and $A_{n\mu}(k)$, with $\mu = x, y$, are respectively the Berry flux and connection for band n. These can be evaluated by calculating the eigenstates of the Hamiltonian presented in supplementary equation 3. Using the numerical method exposed in Ref. 7, we find the Chern number to be zero outside the chiral region ($i.e$ $V_z > \sqrt{\Delta_{\rm S}^2 + \mu^2}$), independently of whether or not we include spin-orbit coupling. This seems to miss the helical structure proposed in the previous section, and in order to get a more detailed glimpse at the topology of the system in the helical case, it is instructive to analyze a simple limiting case: $\Delta_{\rm S} = 0$ and $\alpha = 0$, for which the the Hamiltonian is spin block diagonal

$$H = \begin{pmatrix} H_+ & 0 \\ 0 & H_- \end{pmatrix}, \tag{10}$$

where

$$H_\pm = (\xi_k \pm V_z)\sigma_z + \frac{\Delta_{\rm T}}{k_{\rm F}}(\sigma_x k_y \mp \sigma_y k_x)\,. \tag{11}$$

We can then calculate the Chern number corresponding to each block. We find $C_\pm = \pm 1$, which gives one pair of (quasi-) helical states, or two chiral states at the edges of the sample. Note that the total Chern number of the system is $C = C_+ + C_- = 0$, as found from the full Hamiltonian. Moreover, the time-reversal invariance is broken, and thus these states are inequivalent, i.e. they are not conjugate Kramers pairs. This in turn implies that the edge states can separate spatially, as found in experiment. We note that these two states are only weakly topologically protected, meaning that impurity scattering can result in mixing between them and open a gap at the crossing point. A more complete Hamiltonian (considering finite $\Delta_{\rm S}$ and α) does not alter our conclusions.

SUPPLEMENTARY NOTE 5: TIGHT BINDING ON A CIRCULAR GEOMETRY

In this part we discuss the BdG Hamiltonian and edge states in the circular geometry pertaining to our experiment. Our Hamiltonian in the Nambu spinor basis, $\Psi(\boldsymbol{r}) = [u_\uparrow(\boldsymbol{r}), u_\downarrow(\boldsymbol{r}), v_\downarrow(\boldsymbol{r}), -v_\uparrow(\boldsymbol{r})]$ reads

$$H = \left(-\eta\nabla^2 - \mu\right)\tau_z + V_z(r)\sigma_z + \Delta_{\mathrm{S}}\tau_x + \left(\alpha\tau_z + \frac{\Delta_{\mathrm{T}}}{k_{\mathrm{F}}}\tau_x\right)\left(\sigma_x\hat{p}_y - \sigma_y\hat{p}_x\right), \tag{12}$$

Owing to the circular symmetry of the Zeeman potential, it is convenient to rewrite it in the polar coordinate:

$$\begin{aligned} H = &\left[-\eta\left(\partial_r^2 + \frac{1}{r}\partial_r - \frac{L_z^2}{r^2}\right) - \mu\right]\tau_z + V_z(r)\sigma_z + \Delta_{\mathrm{S}}\tau_x \\ &+ \left(\alpha\tau_z + \frac{\Delta_{\mathrm{T}}}{k_{\mathrm{F}}}\tau_x\right)\begin{pmatrix} 0 & ie^{-i\theta}(-i\partial_r - \frac{1}{r}\partial_\theta) \\ -ie^{i\theta}(-i\partial_r + \frac{1}{r}\partial_\theta) & 0 \end{pmatrix}. \end{aligned} \tag{13}$$

The above Hamiltonian commutes with the operator

$$J_z = L_z + \frac{1}{2}\sigma_z\,. \tag{14}$$

Therefore, we can choose the spinors eigenstates of the Hamiltonian, eigenstates of J_z with the eigenvalue m_J of the form:

$$\Psi_{m_J}(\boldsymbol{r}) = e^{iL_z\theta}\Psi_{m_J}(r) = \begin{pmatrix} u_{\uparrow m_J}(r)e^{i(m_J-1/2)\theta} \\ u_{\downarrow m_J}(r)e^{i(m_J+1/2)\theta} \\ v_{\downarrow m_J}(r)e^{i(m_J-1/2)\theta} \\ -v_{\uparrow m_J}(r)e^{i(m_J+1/2)\theta} \end{pmatrix}, \tag{15}$$

where the spinor $\Psi_{m_J}(r)$ are eigenvectors of the Hamiltonian $\widetilde{H}_{m_J}$ defined as [5]

$$\begin{aligned} \widetilde{H}_{m_J} &= e^{-i(m_J-\sigma_z/2)\theta} H e^{i(m_J-\sigma_z/2)\theta} \\ &= \left[-\eta\left(\partial_r^2 + \frac{1}{r}\partial_r - \frac{(2m_J-\sigma_z)^2}{4r^2}\right) - \mu\right]\tau_z + V_z(r)\sigma_z + \Delta_{\mathrm{S}}\tau_x \\ &+ \left(\alpha\tau_z + \frac{\Delta_{\mathrm{T}}}{k_F}\tau_x\right)\left[\left(\partial_r + \frac{1}{2r}\right)i\sigma_y + \frac{m_J}{r}\sigma_x\right]. \end{aligned} \tag{16}$$

Defining $\hat{k}_\theta = -\frac{i}{r}\partial_\theta$ we can see that for a large r the relation $\hat{k}_\theta\Psi_{mJ}(R) \simeq \frac{m_J}{R}\Psi(R)$ holds. Our aim is to discretize the system in order to compute the dispersion of the edge state. Because the scalar product between the spinor is defined as $\int \psi^*_{m_J}(r)\phi_{m_J}(r)r\mathrm{d}r$, we renormalize our spinors as $\hat{\Psi}_{m_J} = \sqrt{r}\Psi_{m_J}$. The hamiltonian we have to discretize thus reads

$$\hat{H}_{m_J} = \left[-\eta\left(\partial_r^2 - \frac{m_J^2 - m_J\sigma_z}{r^2}\right) - \mu\right]\tau_z + V_z(r)\sigma_z + \Delta_\mathrm{S}\tau_x \\ + \left(\alpha\tau_z + \frac{\Delta_\mathrm{T}}{k_\mathrm{F}}\tau_x\right)\left(i\sigma_y\partial_r + \frac{1}{r}m_J\sigma_x\right). \tag{17}$$

This Hamiltonian is easily discretizable and provides a 1D mapping of our system for every quantum number m_J. Such procedure offers an efficient way to diagonalize the entire 2D Hamiltonian. The results of this tight binding model are presented in supplementary figure 9. On one hand, plots 9**a** and 9**b** are an example of a pure chiral edge state produced without triplet superconductivity ($\Delta_\mathrm{T} = 0$) but with a singlet term together with Rashba and Zeeman terms. In this case, no X shape appears in the LDOS $\rho(E, r)$ (see supplementary figure 9**b**). On the other hand, plots 9**c** and 9**d** represent a realization of a topological transition with a non-zero triplet term together with additionnal terms which values are comparable to the ones used in supplementary figure 9**a** and 9**b**. It is found that as soon as there is a (small) triplet component, the spatial evolution of the LDOS shows helical-like features which resembles the X shape behaviour shown in the main text. In addition the gap is found to be reduced around the critical Zeeman field $V_{z,\mathrm{crit}}$, which we believe to be a numerical artefact due to the impossibility at present to be in the correct physical limit where the cluster size is smaller the coherence length. Supplementary figure 9**c** shows that there is a single chiral edge state. We nevertheless believe that a reminiscence of the helical state (for $V_{z,\mathrm{crit}} = 0$) will survive in the correct physical limit and will contribute to the X shape spatial dispersion of $\rho(E, r)$, as we observe in the experimental results. This is supported by a more sophisticated theoretical approach that we are currently developping. This approach is based on exact low-energy Bessel functions for a circular geometry including triplet superconductivity and a strong and sharp Zeeman field. Note that with respect to the simulations shown in Fig.3 of the main text, the spatial profile of the Zeeman field used in supplementary figure 9**a-d** is sharper.

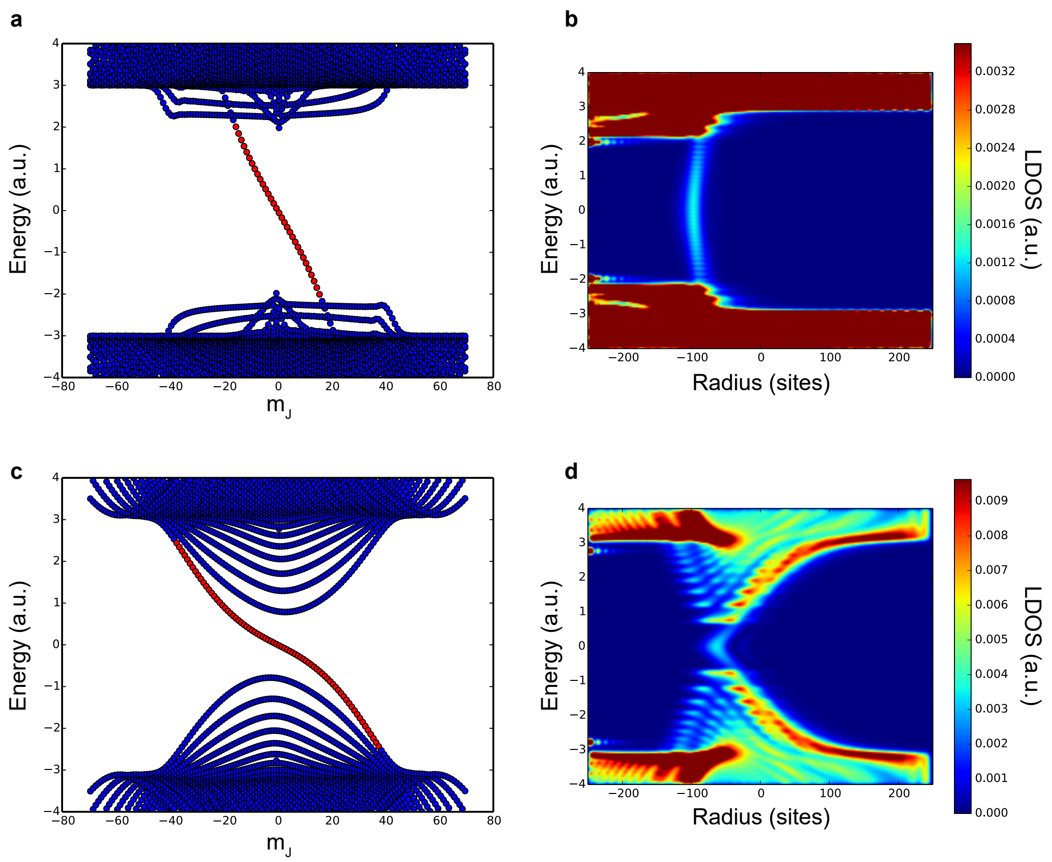

Supplementary Figure 9: **Tight binding calculation in a disk geometry - a** dispersion and **b** LDOS of a cluster without triplet superconductivity with a singlet term together with Rashba and Zeeman terms. The tight binding parameters are $t = 50$, $\mu = 5$, $V_{z,\mathrm{max}} = 8$, $\Delta_{\mathrm{S}} = 3$ and $\alpha/k_{\mathrm{F}} = 4.5$; (**c**) dispersion and (**d**) LDOS of a cluster with triplet superconductivity and other similar parameters. The tight binding parameters are $t = 50$, $\mu = 5$, $V_{z,\mathrm{max}} = 12$, $\Delta_S = 4$, $\Delta_{\mathrm{T}} = 1.4$ and $\alpha/k_{\mathrm{F}} = 2.2$.

SUPPLEMENTARY NOTE 6: CHARACTERIZING AND IMAGING THE BURIED CO-SI ISLANDS

A. Electronic properties of the buried Co-Si islands

On one hand it seems rather puzzling that the buried Co-Si islands are not visible in STM topography measurements in the energy range [-2;+2] V. On the other hand we provide below two different experimental techniques, TEM and STM, that enabled us to image the buried Co-Si islands and extract their size. The extracted size is in good agreement with

the size of the spectroscopic features seen in Fig.2 of the main text and supplementary figure 5 of the supplementary part, i.e. of few nanometers of lateral size. We interpret the fact that the buried Co-Si islands do not contribute, or negligibly, to the local DOS of the top Pb monolayer as being associated with their (almost) insulating state. This is further supported by the observation that these buried Co-Si islands do not reduce locally the superconducting gap of the SIC monolayer by inverse proximity effect, as it should if the islands would have some metallic properties [8]. As a direct consequence, because the buried Co-Si islands are (almost) insulating but provide a suitable large exchange field leading to local topological superconductivity above them, it implies that the Co-Si islands are insulating ferromagnets. This is in agreement with ab initio spin-polarized DFT calculations of Co-Si cluster having different Co concentration (see Ref 28 of the main text: Chulsu et al Journal of Magnetic materials 306, 156, 2006 : Magnetic properties of CoâĂŞSi alloy clusters). All Co-Si clusters are theoretically found to be magnetic.

B. Structure of the buried Co-Si islands

The early stages of growth of Co on Si(111)-7×7 has been widely studied because of the importance of controlling silicide formation for silicon-metal contacts in microelectronics [9–11]. When Co, in the range below 4 ML, is deposited at room temperature no epitaxial cobalt silicide is formed [12–14]. When the sample is further annealed above 350° two-dimensionnal $CoSi_2$ epitaxial islands and layers start to form in a layer-by-layer way [12–14]. Nevertheless X-ray grazing incidence diffraction experiments reveal that the planar $CoSi_2$ films are rich in defects, Co occupying various sites. When the sample are further annealed above 500° three-dimensionnal epitaxial $CoSi_2$ islands of good quality form [12–14].

In our case the sample preparation is done in the following way: we first deposit $10^{-}3$ ML Co on the Si(111)-7×7 surface at room temperature, then deposit 4 ML Pb also at room temperature. Further on we anneal the whole sample at 375° for 90 seconds. From the previous paragraph we infer that our annealing temperature is above the threshold for planar $CoSi_2$ island formation. Thus we expect that Co atoms will diffuse through the Si surface and that small defected $CoSi_2$ islands will be energetically able to form below the Pb layer. We were further able to image the buried Co-Si islands, which we present below.

C. Imaging the buried Co-Si islands

We succeeded to image the buried Co-Si islands by direct bright-field TEM experiments with a JEOL 2100F microscope working at 200 keV. A plan-view TEM image is shown in supplementary figure 10. When trying to zoom on the Co-Si particles, the contrast diminishes and we cannot image them anymore. Using X-ray Energy Dispersive Spectroscopy (XEDS), we could measure only the Pb and Si signal, but not the Co one due to its very-low concentration (below 1 percent). We also tried to perform Scanning TEM-HAADF experiment (in dark-field) on a TITAN Themis 200 microscope. The 200keV highly convergent electron beam made the single Co-Si particles to fade away and disappear, probably destroying them, contrary to the classical TEM which uses a parallel beam and enabled us to image the particles as shown in supplementary figure 10. This unfortunately prevented us performing a local chemical analysis of single Co-Si particle.

We could also reveal the buried Co-Si islands and image them with STM. To do so, we performed an annealing of one sample during 2 hours at 400°C, which is long enough to desorb most of the Pb atoms from the SIC monolayer, and obtained the structure presented in Fig.1b of the main text. Most of the Pb atoms then desorb from the surface, except for less than about 1/6 ML [15]. The phase we obtained is highly disordered and has some similarities with a phase reported in the literature as the mosaic phase [16]. Additionally, we observed only a few remaining islands that appear as bright spots on this figure, separated by 50 nm in average (some of them are indicated by white arrows on the figure). The step edges have been strongly modified during the annealing and appear shredded indicating that the Si atoms are also moving during this procedure.

The islands left on the sample are interpreted as the buried Co-Si islands. The islands remain most probably capped with Pb as their height is of a few Angstroms above the remaining surface plane which consists in intermixed Pb and Si atoms [16]. The inset of Fig.1b presents a close-up topography of such an island where we adapted the color code in order to see both the surface of the sample and the island shape. While we still have a global truncated triangular shape characteristic of Pb islands we also see on top of the island a quasi-circular contour that is not seen in the case of Pb islands alone. The characteristic lateral size of the Co-Si islands seen in Fig.1b is around 5-10 nm in very good agreement with the radius of the topological edge states measured at zero-energy shown in Fig.1d of the main text.

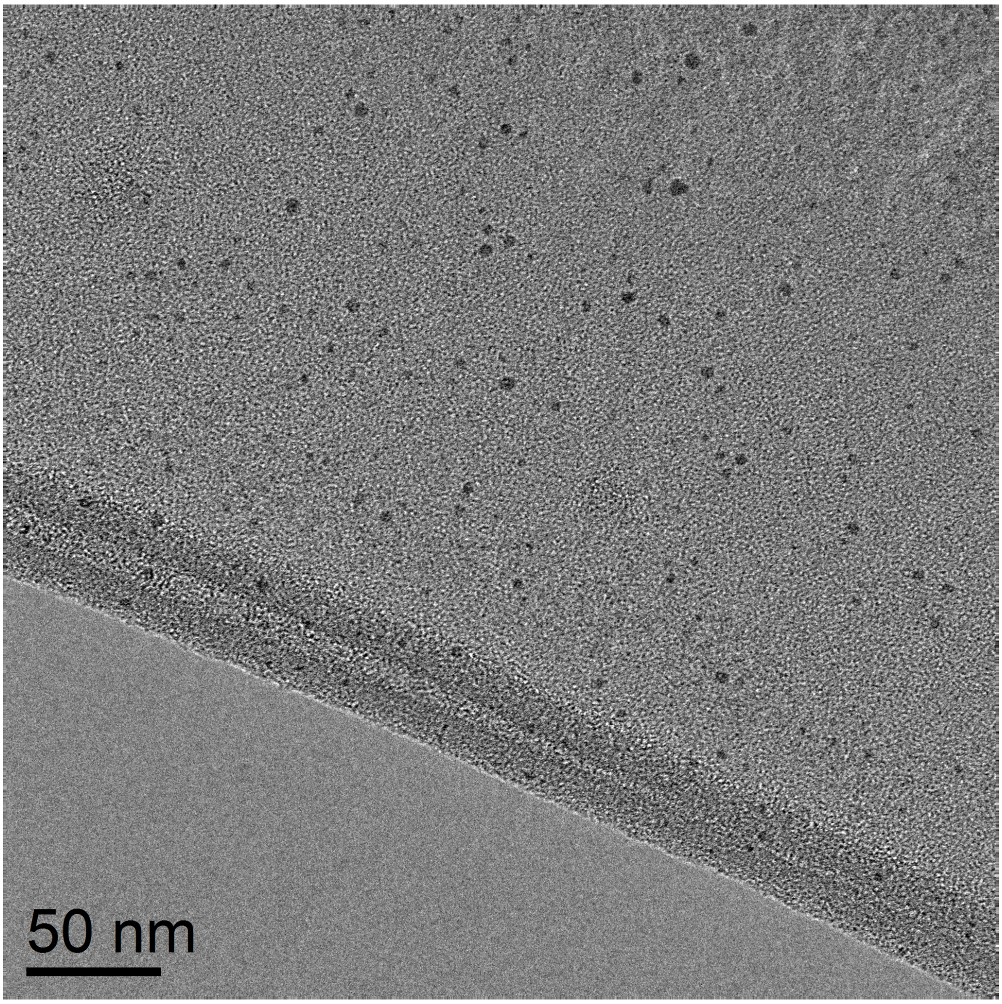

Supplementary Figure 10: **Plan-view TEM image revealing the buried Co-Si clusters** - The Co-Si buried clusters are seen as darker regions. Their size ranges from few nm to less than 10 nm for the largest.

SUPPLEMENTARY NOTE 7: AN EXAMPLE OF A NON-DISPERSIVE YU-SHIBA-RUSINOV BOUND STATE AROUND A SINGLE MAGNETIC ATOM

Occasionally, In addition to the dispersive edge states around magnetic domain we also encounter some bound states around single magnetic impurities (probably caused by single atomic Co impurities). These bound states are the so-called Yu-Shiba-Rusinov bound states (YSR). They manifest in the gap as two peaks whose energy position don't change as function of the distance to the impurity as shown on supplementary figure 11 (Note that this measurement was done with a normal Pt tip). It was recently shown that in a two-

dimensional material such as 2H-$NbSe_2$ the spatial extent of the YSR bound states was quite large due to a dimensionality effect [17]. We indeed confirm this effect here since we find that the bound state induced by a single Co impurity can still be seen in the gap several tens of nanometers away from the magnetic atom. In comparison, in bulk lead, YSR states are no more visible beyond one nanometer from the impurity [18].

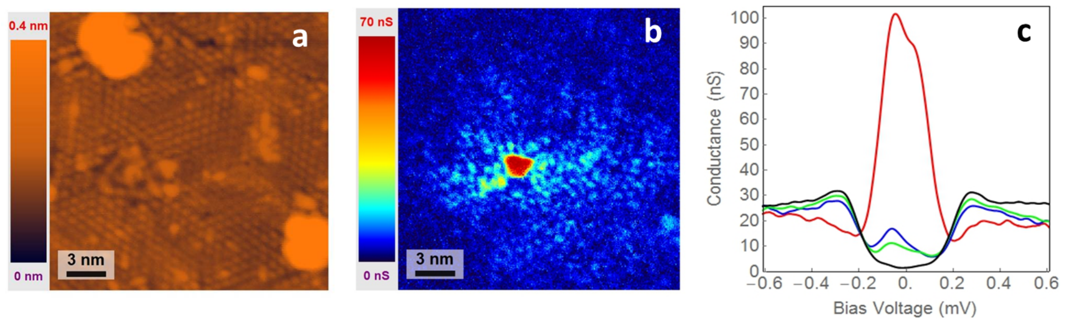

Supplementary Figure 11: **A Yu-Shiba-Rusinov bound state around a single magnetic impurity - a**. Topography of a striped incommensurate Pb/Si(111) sample with a non superconducting PtIr tip. **b**. Conductance map measured at E_F showing a Yu-Shiba-Rusinov bound state localized around a magnetic impurity. **c**. Tunneling spectra taken on top of the impurity (red), at 5 nm (blue), 10 nm (green) and far from the impurity (black).

The energy and spatial pattern of the YSR bound states is at odds with the dispersive behavior of the edge states that we report in this paper. The YSR states are non-dispersive, their energy remains constant, whatever the distance from the impurity. Moreover, as can be seen on supplementary figure 11, the YSR bound states give a very strong signal when measuring the LDOS right on top of the magnetic impurity and their amplitude typically decays as $1/\sqrt{r}\exp(-r/\xi)$. The behavior of the YSR bound states is very different from the one of the dispersive states that are strongly localized at the edge of the magnetic island at $E \approx E_F$ and appear as a thin line of width $w \approx \lambda_F \ll \xi$ ($w \approx 0.5$ nm $\ll 50$ nm). The striped incommensurate Pb/Si(111) monolayer is quite disordered at the atomic scale. As a consequence the YSR wavefunction appears as an irregular speckle like pattern (see supplementary figure 11). When the 2D system has a crystalline structure like for example in 2H-$NbSe_2$, the YSR wavefunction reflects the auto-correlation of the Fermi surface of the material [17]. By contrast, the dispersive states appear as quite regular with a well defined

ring shaped structure close to E_F (see Figs. 1e-2b of the main text, and supplementary figure 5b). However, for energies close to the gap edge the dispersive states starts to be affected by the disorder (see Fig. 2d and supplementary figure 5d).

SUPPLEMENTARY NOTE 8 : SPECTROSCOPIC IMAGING OF MAJORANA DISPERSION WITH A NORMAL PT TIP

The energy resolution of an STM probe with a normal tip is $3.5k_\mathrm{B}T$. Thus for a measurement temperature of 300 mK, the energy resolution is at best 90 μeV. This has to be compared to the upper limit resolution of 20 μeV obtained with a Pb superconducting tip. However, even with a normal tip with poor resolution, the dispersive states that appear around topological domains can still be measured. Supplementary figure 12.a shows a large scale topography with two terraces of Pb/Si(111). An atomically resolved image of the lower terrace is shown on supplementary figure 12.c. As can be seen, the Pb monolayer is in the devil's staircase phase which is a mixture of some linear shaped $\sqrt{7}\times\sqrt{3}$ domains coexisting at the nanometer scale with some striped incommensurate domains [19]. The estimated Pb coverage is 1.26-1.28 monolayer. This sample is again different from all the ones discussed previously.

Supplementary figure 12.b is a map of the tunneling conductance at the Fermi level ($V = 0$ mV) with a normal tip. Two separate ring like structures are observed at the Fermi level in the top left and bottom left part of the image. A zoom of the lower ring-shaped structure is shown on supplementary figure 12.d. Unfortunately only a part of the ring was measured but this doesn't prevent us from analyzing the spectroscopic features of this object. A tunneling spectrum measured far from the ring, at the lower left corner of the image 12d, is shown in blue in supplementary figure 12.e. This spectrum exhibits a well defined gap around the Fermi level with zero-conductance at E_F. A spectrum measured on the ring-shaped feature observed at zero bias is shown in red: it exhibits a clear peak centered at the Fermi level. Due to the thermal resolution of 90 μeV, the peak is quite broadened compared to the one obtained by deconvolution of SIS spectra using a superconducting tip at the same temperature (see inset of figure 2e of main text).

A radial profile from the center of the ring-shape structure to 25 nm away is shown on 12f. Topological edge states closing the gap at the edge of the topological domain are

measured, but the bad spectral resolution doesn't lead to results as clear as the ones shown in spectroscopic maps performed with a Pb superconducting tip.

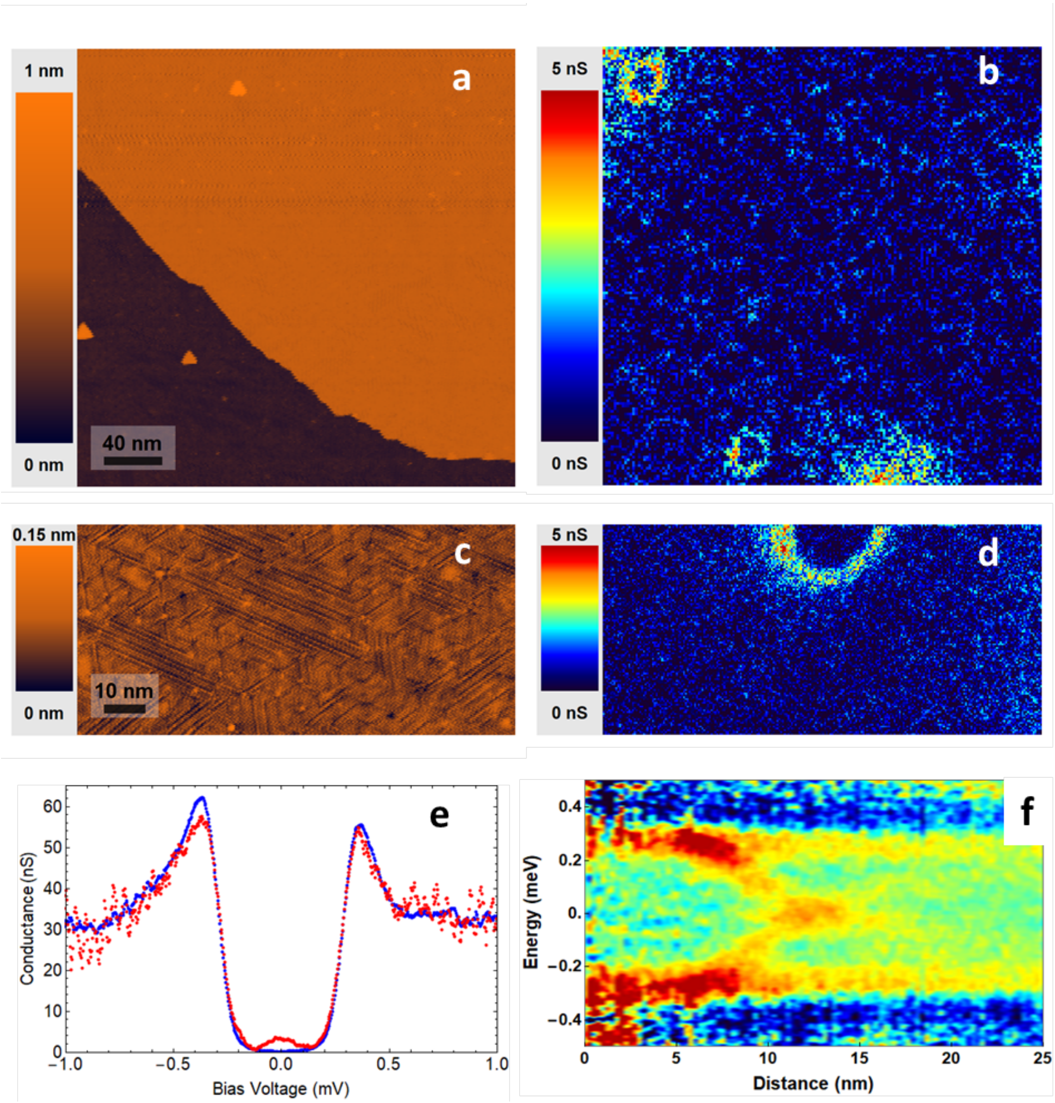

Supplementary Figure 12: **Majorana dispersion observed with a normal PtIr tip at 300 mK** - **a** STM topography of a Pb/Si(111) monolayer with an approximate coverage of 1.26-1.28 monolayer. In **b** conductance map at zero bias of the area shown in **a**, it exhibits two ring-shaped structures in the top-left and lower-left corners. **c-d**, zoom-in of the previous images over the lower ring-shaped spectral feature. **e**, dI/dV tunneling spectra measured far from the ring (blue) and on top of the ring (red). In **f** line cut from the center of the ring to 25 nm away, showing topological edge states dispersing in real space throughout the sample superconducting gap, for clarity a reference spectrum (blue curve in **e**) was subtracted in order to enhance the contrast.

SUPPLEMENTARY REFERENCES

[1] L. Serrier-Garcia, J. C. Cuevas, T. Cren, C. Brun, V. Cherkez, F. Debontridder, D. Fokin, F. S. Bergeret, and D. Roditchev, *Scanning tunneling spectroscopy study of the proximity effect in a disordered two-dimensional metal* Phys. Rev. Lett. 110, 157003 (2013).

[2] Christian R. Ast, Berthold Jäck, Jacob Senkpiel, Matthias Eltschka, Markus Etzkorn, Joachim Ankerhold and Klaus Kern, *Sensing the quantum limit in scanning tunnelling spectroscopy*, Nat. Comm. **7**, 13009 (2016)

[3] Ivar Giaver, *Electron tunneling between two superconductors*, Phys. Rev. Lett. **5**, 464 (1960)

[4] M. Ruby, B.W. Heinrich, J.I. Pascual and K.J. Franke, *Experimental demonstration of a two-band superconducting state for lead using scanning tunneling spectroscopy*, Phys. Rev. Lett. **114**, 157001 (2015)

[5] J. D. Sau, S. Tewari, R. M. Lutchyn, T. D. Stanescu, S. Das Sarma *Non-Abelian quantum order in spin-orbit-coupled semiconductors: Search for topological Majorana particles in solid-state systems*, Phys. Rev. B, **82** (21), 214509. (2010).

[6] L. P. Gorḱov and E. I. Rashba *Superconducting 2D system with lifted spin degeneracy: Mixed singlet-triplet state*, Phys. Rev. Lett., **87**(3), 037004 (2001).

[7] T. Fukui, Y. Hatsugai and H. Suzuki *Chern numbers in discretized brillouin zone: efficient method of computing (spin) hall conductances*, J. Phys. Soc. Japan, **74** (6), 1674-1677 (2005).

[8] W.L. McMillan *Tunneling Model of the Superconducting Proximity Effect*, Phys. Rev., **175**, 537 (1968).

[9] S.C. Wu *et al. Structural reactions of Si(111) with Co and formation of cobalt silicide*, Phys. Rev., B **33**, 2900 (1986).

[10] D.R. Hamann *New silicide interface model from structural energy calculations*, Phys. Rev. Lett., **60**, 313 (1988).

[11] P.A. Bennett *et al. Ring clusters in transition-metal-silicon surface structures*, Phys. Rev. Lett., **69**, 1224 (1992).

[12] C. Pirri *et al. Surface structure of epitaxial CoSi2 crystals grown on Si(111*, Phys. Rev. B, **33**, 4108 (1986).

[13] J. Zegenhagen *et al. Co on Si(111: siliced formation)*, Phys. Rev. B, **44**, 13626 (1991).

[14] M. Loffler *et al. Pulsed laser deposition of Co and growth of* $CoSi_2$ *on Si(111)*, Appl. Phys. A, **81**, 1651 (2005).

[15] J.A. Carlisle *et al. Photoemission study of the growth, desorption, Schottky-barrier formation, and atomic structure of Pb on Si(111)*, Phys. Rev. B, **45**, 3400 (1992).

[16] B. Ressel, J. Slezák, K. C. Prince and V. Cháb *Quantized valence states of the Pb/Si(111) mosaic phase*, Phys. Rev. B, **66**, 035325 (2002).

[17] G. C. Ménard, S. Guissart, C. Brun, S. Pons, V. S. Stolyarov, F. Debontridder, M. V. Leclerc, E. Janod, L. Cario, D. Roditchev, P. Simon and T. Cren, *Coherent long-range magnetic bound states in a superconductor*, Nature Physics **11**, 1013-1017 (2015)

[18] S.H. Ji et al., *High-resolution scanning tunneling spectroscopy of magnetic impurity induced bound states in the superconducting gap of Pb thin films*, Phys. Rev. Lett. 100, 226801 (2008).

[19] M. Hupalo J. Schmalian and M.C. Tringides, *Devil's staircase in Pb/Si(111) ordered phases*, Phys. Rev. Lett 90, 216106 (2003).
